# Welcome to the New Journal *Non-Coding RNA*!

**DOI:** 10.3390/ncrna1010001

**Published:** 2014-11-21

**Authors:** George A. Calin, Franck Vazquez

**Affiliations:** 1 Department of Experimental Therapeutics, Unit 1950, Center for RNA Interference and Non-Coding RNAs, The University of Texas, MD Anderson Cancer Center, 1881 East Rd, Unit 1950, 3SCR4.3424, 301429, Houston, TX 77030, USA; 2 Managing Editor of *Non-Coding RNA*, MDPI AG, Klybeckstrassse 64, CH-4057 Basel, Switzerland

**Keywords:** microRNA, siRNA, piRNA, esiRNA, lincRNA, non-coding RNA, AGO, PIWI, DICER

Dear colleagues and non-coding RNAs aficionados,

You will probably ask ‘why a new journal’?, these days when we are flooded not only by information of any type in any sector of our life, but also by so many new journals that come and disappear like comets in the summer sky! The answer is simple: because we believe that this field finally deserves to have a dedicated journal where its wide community will be able to communicate and exchange its latest findings in one centralized place. Moreover, with your outstanding contributions and by publishing papers that promulgate the ‘thinking out of the box’, we will be able to build a reputation for *Non-Coding RNA* to survive over the years to come. 

The journal will definitely benefit of his extremely dynamic community which studies non-coding RNA genes that do not encode for proteins but regulate their function. This year about ten thousand articles were published in this field. 

This field achieved maturity, but a lot remains to be discovered! The first discovered microRNA, lin-4, was identified already more than 20 years ago [1], but for 7 years, nobody understood if this class of genes is a worm-related story or if it has wider implications. Since 2001 and the discovery of multiple microRNAs in various organisms [2,3,4], followed by the elucidation of the small RNA component of a plant genome in 2005 [5], long and short non-coding RNAs have gained increasingly more attention, particularly for their central roles in development, in defence, or in genome maintenance [6,7,8,9,10]. Many different types of non-coding RNAs have already been described, like endo-siRNAs, scnRNAs, ta-siRNAs, piRNAs, ceRNAs, lincRNAs [6,7,8,9,10], … and many more might well pop-up very soon. Non-coding RNAs have also more recently emerged as biomarkers to diagnose and treat cancers [11]. Recently, many translational approaches have been initiated. 

This field has been built stone after stone from the many scientific contributions from extremely diverse horizons, studying gene silencing in plants, position effect variegation in drosophila or quelling in fungi. *Non-Coding RNA* will definitely be the place for publication of findings made in any model organisms. We know how important it is to consolidate our scientific efforts in this field!

Our aim is to publish manuscripts that will have a high impact on the future development of the field! We will publish articles on topics as varied as RNA biology and processing, translational and clinical studies involving short and long non-coding RNAs, as well as technological developments. Not only this, but *Non-Coding RNA* will house opinions, concepts and ideas that are at the cutting edge of the field and would have difficulty finding a place in other journals. We all know that truly novel discoveries have difficulty finding the right judges/reviewers, as well as the right journals, to be published without delays and endless debates. We can assure you, the authors, the readers and the members of the Editorial Board, that *Non-Coding RNA* will be a place where good science will find support and a nest to develop in further publications that will impact a field that is the new Eldorado of science––the study of short and long non-coding RNAs.

Welcome to all of you and let’s work altogether as a team bound by one magic trait––the love to do our hobby, discoveries related to non-coding RNAs. Good luck to all of us on this endeavor, let’s have fun and wish *Non-Coding RNA* a long and rewarding life!


George and Franck

